# Probiotic Consumption Boosts Thymus in Obesity and Senescence Mouse Models

**DOI:** 10.3390/nu14030616

**Published:** 2022-01-30

**Authors:** Florencia Balcells, María José Martínez Monteros, Alba Lorena Gómez, Silvia Inés Cazorla, Gabriela Perdigón, Carolina Maldonado-Galdeano

**Affiliations:** 1 Laboratorio de Inmunología, Centro de Referencia para Lactobacilos (CERELA-CONICET), San Miguel de Tucumán 4000, Argentina; fbalcells@cerela.org.ar (F.B.); mjmartinez@cerela.org.ar (M.J.M.M.); scazorla@cerela.org.ar (S.I.C.); perdigon@cerela.org.ar (G.P.); 2 PatLab Laboratorio de Anatomía Patológica Citopatología e Inmunohistoquímica, San Miguel de Tucumán 4000, Argentina; albalgomez@yahoo.com.ar; 3 Cátedra de Inmunología, Facultad de Bioquímica, Química y Farmacia, Universidad Nacional de Tucumán, San Miguel de Tucumán 4000, Argentina

**Keywords:** thymus, obesity, aging, immune system, probiotics, probiotic cell wall

## Abstract

The ability of the immune system to respond to different pathogens throughout life requires the constant production and selection of T cells in the thymus. This immune organ is very sensitive to age, infectious processes and nutrition disorders (obesity and malnutrition). Several studies have shown that the incorporation of some probiotic bacteria or probiotic fermented milk in the diet has beneficial effects, not only at the intestinal level but also on distant mucosal tissues, improving the architecture of the thymus in a malnutrition model. The aim of the present study was to determine whether supplementation with the probiotic strain *Lactobacillus casei* CRL 431 and/or its cell wall could improve body weight, intestinal microbiota and thymus structure and function in both obese and aging mice. We evaluated probiotic administration to BALB/c mice in 2 experimental mouse models: obesity and senescence, including mice of different ages (21, 28, 45, 90 and 180 days). Changes in thymus size and histology were recorded. T-lymphocyte population and cytokine production were also determined. The consumption of probiotics improved the cortical/medullary ratio, the production and regulation of cytokines and the recovery of mature T-lymphocyte populations of the thymus in obese and old mice. Probiotic incorporation into the diet could not only modulate the immune system but also lead to thymus function recovery, thus improving quality of life.

## 1. Introduction

The thymus is a primary lymphoid organ present in all vertebrates [1] and is also responsible for the development of self-restricted, self-tolerant and immunocompetent T cells. The maturation process of T lymphocytes involves different stages of proliferation and differentiation that depend on the instructions from the specialized thymus microenvironment [2]. T lymphocytes express the TCR receptor for the recognition of antigens with different antigenic specificities. Thymocytes that have a TCR with specificity for their own antigens (self-reactive lymphocytes) are negatively selected and eliminated by apoptosis. The lymphocytes that have a TCR for foreign antigens are positively selected and differentiated into functionally mature LTCD4+ or LTCD8+ lymphocytes that are finally overturned into the systemic circulation [3,4,5,6]. The positive selection induced in the thymic cortex also promotes the migration of positively selected thymocytes to the medullary region. This migration of thymocytes is driven by chemokines produced mainly by the epithelial cells of the thymus, with chemokine CCRL7 being essential in this process [7,8,9,10].

It is well-defined and accepted that the thymus undergoes a regression with age, affecting its functional capacity and size. However, little is known about the mechanisms concerning thymic involution. This process is accompanied by increased sensitivity to cancer, infectious diseases and autoimmune diseases and is related in part to diabetes, obesity, and hypertension. These changes are associated with a decline in the normal function of the immune system, a process described by the term immunosenescence, with a progressive deterioration in the ability to respond against new antigens [11,12].

The mechanisms involved in this process are not fully understood, but an important cause or effect is the gradual decrease in thymus size and the replacement by fat in early adulthood [13,14,15,16,17]. In this sense, studies in obese mice reported many alterations of the thymus with increased perithymic adipose tissue and a reduction of the total number of thymocytes, similar to the immunosenescence process that takes place during aging [18]. 

In this sense, studies in obese mouse models (Lep^ob^/Lep^ob^) showed evident thymic involution and revealed the important role of leptin in its function [19]. Other authors reported a correlation between obesity and thymic aging which affected thymopoiesis and immune surveillance, suggesting that obesity accelerates thymic involution and represents a potent modifier of immunosenescence mechanisms [20]. Actually, there are studies in humans that indicate a relationship between obesity and involution of the thymus gland [21]. 

In this respect, considering previous results observed in malnourished mice in which probiotic supplementation improved body weight and the function of the immune system at the systemic and intestinal levels, as well as regarding the histology and function of the thymus [22,23], we propose probiotics as an adjuvant of the immune system in a malnutrition process. 

Nowadays, the role of probiotics on health, especially lactic acid bacteria, is being studied by researchers worldwide [24]. In our laboratory, we have demonstrated their positive effects on the immune system [25,26,27,28,29,30,31,32,33].

In vitro studies have shown that several lactic acid bacterial (LAB) strains elicit an immunomodulatory effect on cells of the innate immune system, including macrophages [34]. 

Another study demonstrated that probiotic cell walls (CWs) activate intestinal epithelial cells (IECs), the clonal expansion of the IgA B-cell population, and the cytokine release that modulates immune cells distant from the gastrointestinal tract. These findings suggest that the CWs of probiotic bacteria could be used as mucosal adjuvants [35].

However, there are no studies showing the effect of probiotic lactic acid bacteria and/or their cell walls on the function and size of the thymus as a result of a severe affection of this organ, such as by obesity and aging disorders. 

The aim of the present study was to determine whether supplementation with the probiotic strain *Lactobacillus casei* CRL 431 and/or with its cell wall could improve the function of the thymus in both obese and aging mouse models.

## 2. Materials and Methods

### 2.1. Bacterial Strain and Culture Conditions

*L. casei* CRL 431 was provided by the CERELA Culture Collection (San Miguel de Tucumán, Argentina). Overnight cultures were grown at 37 °C in 5 mL of sterile Mann-Rogosa-Sharp (MRS) broth (Britania, Buenos Aires, Argentina), as previously described by Novotny Núñez et al. [22]. The final concentration of probiotic bacteria was 2 ± 1 × 10^8^ CFU/mL, an optimal dose for the activation of the intestinal immune system for this strain [23].

### 2.2. Isolation of the Cell Wall of a Probiotic Bacterium

The cell wall (CW) of the probiotic bacterium *L. casei* CRL 431 was purified as previously described by Lemme Dumit et al. [34]. Briefly, the probiotic bacterium was grown in MRS broth for 16 h at 37 °C, and the biomass was harvested at 9900× *g* for 10 min at 4 °C and washed three times with distilled sterile cold water. The pellet was lysed three times using a French press at 20,000 psi.

The product obtained was centrifuged at 4000× *g* for 15 min at 4 °C. The supernatant obtained was harvested at 30,000× *g* for 30 min at 4 °C. The pellet was delipidated and treated with DNase I from bovine pancreas (Sigma-Aldrich, St. Louis, MO, USA) (50 µg/mL) and ribonuclease A (Sigma Aldrich) (100 µg/mL) at 37 °C with stirring for 4 h. The insoluble product was washed with distilled sterile water and frozen at −80 °C; the products obtained were used as cell walls (CW) from *L. casei* CRL431.

### 2.3. Animals, Feeding Procedures and Study Design for Obese and Aging Experimental Mouse Models

Male BALB/c mice were provided by CERELA (San Miguel de Tucuman, Argentina) from a closed random-bred colony. 

For the senescence studies, a batch of 30 21-day-old mice was used. The batch was divided into 2 groups according to the diet received during the experience (180 days): 

Normal control (NC) group: animals were fed *ad libitum* with conventional food and water.

Lc431 group (Lc): animals received conventional food and supplementation with the probiotic bacterium in their drinking water.

Three animals of each group (NC and Lc) were sacrificed by cervical dislocation at different times (21, 28, 45, 90 or 180 days) (Figure S1A). 

For the studies performed in the obesity model, a batch of 20 21-day-old mice was divided into 2 groups: normal control (NC), which were fed ad libitum with conventional balanced food, and obese control (OC), which were fed a high-fat diet (HFD). The HFD was made as previously described by Balcells et al. [33]. Each group (NC and OC) was subdivided into sub-groups according to the dietary supplement administered, as follows:

1. Normal control (NC): animals fed *ad libitum* with conventional food and water;
  1.2. Normal control mice supplemented with probiotics (NC+P): mice received a conventional diet and a suspension of the probiotic bacterium in the drinking water;
2. Obese control (OC): animals fed *ad libitum* with the HFD and water;
  2.1. Obese plus probiotics (OC+P): OC animals received a HFD and a suspension containing probiotic bacteria. See details in Figure S1B.

The drinking volume consumed was measured daily in each cage, considering the number of mice per cage. Each animal consumed about 5–6 mL of liquid per day. The bottles containing diet supplements were replaced daily to maintain their quality.

After 12 weeks of feeding, mice from each group were sacrificed by cervical dislocation.

The samples collected in all the experimental groups included serum, small intestine and small intestinal fluid, large intestine, thymus and spleen.

Mice were maintained in a room with 12-h light/dark cycles at 20 ± 2°C and were weighed three times per week, for 12 weeks. The thymus weight was taken the day of the sacrifice and expressed as the ratio between body weight/thymus weight.

All animal protocols were preapproved by the CERELA Animal Protection Committee (CRL-BIOT-LI-2011/1A) and conducted in accordance with the guidelines established by the Consejo Nacional de Investigaciones Científicas y Técnicas (CONICET). 

### 2.4. Serum and Small Intestinal Fluid Samples

The mice were anesthetized with ketamine-xylazine, and blood was extracted from each one by cardiac puncture and collected in Eppendorf tubes. Immediately, the blood samples were centrifuged at 5000× *g*, and the serum was stored at −20 °C until use.

Small intestinal fluid was obtained by washing the intestine (duodenum, jejunum and ileum) with 1 mL of PBS and immediately centrifuged at 5000× *g* for 15 min at 4 °C. The supernatant was separated and stored at −20 °C.

### 2.5. Analysis of Intestinal Microbiota

The large intestines were aseptically removed at the time of sacrifice according to each experimental model: 12 weeks for the obesity mouse model and at 21, 28, 45, 90 or 180 days for the senescence mouse model. The large intestines were weighed and placed in sterile tubes containing 5 mL of peptone water (0.1%) and were immediately homogenized under sterile conditions. We performed serial dilutions that were spread on specialized agarised media for Enterobacteria, Lactobacilli and total anaerobes, as previously described [30].

### 2.6. Histological Studies

The thymus was removed and fixed with 10% formaldehyde in PBS solution (pH 7). After fixation, the tissues were dehydrated and embedded in paraffin. Serial paraffin sections (4 mm thick) were cut and used for hematoxylin–eosin staining.

The cortical/medullary ratio was determined by measuring the length of the thymus cortex and medullary area of the thymic lobules using Axiovision software, which allows photographs taken under the microscope to be analyzed. The results were expressed as the relationship between the length of the cortex and the length of the medulla in mm.

### 2.7. Determination of T-Cell Population in the Thymus

The thymus was removed aseptically and collected in tubes containing 3 mL of RPMI-1640 medium supplemented with 10% bovine fetal serum (FBS), disintegrated and centrifuged at 1000× *g* for 10 min. The pellets were resuspended in 2 mL of RPMI. The final concentration was adjusted to 3 × 10^6^ cell/mL and incubated in the dark at 4 °C for 1 h with the antibodies APC hamster anti-mouse CD3 (BD Pharmigen, Catalog No. 561826), FITC rat anti-mouse CD4 (BD Pharmigen, Catalog No. 557307) and PerCP rat anti-mouse CD8 (BD Pharmigen, Catalog No. 553036). After resuspension in 500 μL of PBS, cells were analyzed using a FACS Calibur flow cytometer (BD Bioscience, San Diego, CA, USA) equipped with an excitation laser source at 488 nm and 635 nm. Samples were run through the Flow cytometer, and 500,000 events were analyzed for each sample using FlowJo 7.6.2 software. 

### 2.8. Ex Vivo Assays in Cell Cultures

The thymus and spleen collected in 5 mL of RPMI-1640 medium (Sigma Aldrich) containing 10% fetal bovine serum (FBS) and gentamicin were mechanically disaggregated using metal meshes. 

Cells of the thymus and spleen were harvested by centrifugation at 10,000× *g* for 15 min at 4 °C. Pellets were resuspended in 2 mL of red blood cell lysis buffer and mixed softly. The reaction was stopped with 8 mL of sterile PBS, and cells were harvested by centrifugation at 10,000× *g* for 15 min. The product obtained was resuspended on 3 mL of RPMI-FBS and adjusted at 7 × 10^6^ cells/mL.

Cells from the obese control group were cultured overnight at 37 °C, 5% CO_2_ in 6-well sterile plates (JET Biofil, Guangzhou, China) in the presence of the complete probiotic bacteria (B) or their CW (W).

For the senescence studies, cells (7 × 10^6^ cells/mL) of the thymus and spleen belonging to mice of different ages (21, 28, 45, 90 and 180 days old) were plated in 6-well sterile plates. We added complete probiotic bacteria or their CW and incubated them overnight at 37 °C; 5% CO2.

The samples were divided into three groups:

1. Normal controls of different ages;
2. Normal controls of different ages with live probiotic bacteria (NC+B);
3. Normal controls of different ages with probiotic cell wall (NC+W).

The supernatants were collected from each well of plates in tubes and centrifuged at 1000× *g* for 15 min. The obtained supernatants were stored in sterile tubes and frozen at −80 °C.

### 2.9. Determination of Cytokine Production 

Interleukin (IL)-6, IL-10, IL-3, IL-12(p70), interferon-gamma (IFN-γ), TFN-α and IL-3 were determined in serum, small intestinal fluids and splenocyte supernatant cultures by enzyme-linked immunosorbent assay, according to the manufacturer’s instructions (BD OptEIA; BD Biosciences, USA catalog numbers: IL-6: 555240; IL-10: 555252; IL-3: 555228; IL-12 (p70): 555258; TNF-α: 555268 and IFN-γ: 555138).

The IL-7 level secreted by thymocytes was determined using the Mouse IL-7 ELISA KIT (Product number: RAB0317, Sigma-Aldrich, St. Louis, MO, USA), following the manufacturer’s instructions. 

The results were expressed as the concentration of each cytokine (pg/mL) of each fluid.

### 2.10. Statistical Analysis

Statistical analyses were performed by GraphPad Prism 6.01 software (GraphPad Software, La Jolla, CA, USA) using the ANOVA general linear model, followed by Tukey’s post hoc test, and *p* < 0.05 was considered statistically significant. All data were expressed as the mean ± standard deviation (SD).

## 3. Results

### 3.1. Changes in Body Weight and Thymus. Effects of Probiotic Supplementation on These Parameters in Obese and Senescent Mice

Feeding mice an HFD induced an increase in the body weight of obese control mice, with their weight being significantly different from the normal control mice at week 9, where the OC mice reached the peak of their body weight.

In contrast, we observed a significant reduction in body weight in the mice that received the HFD and the supplementation with the probiotic strain suspension at week 10 compared with the OC mice (*** *p* < 0.001), being a little lower than the normal control. 

**Figure 1:**
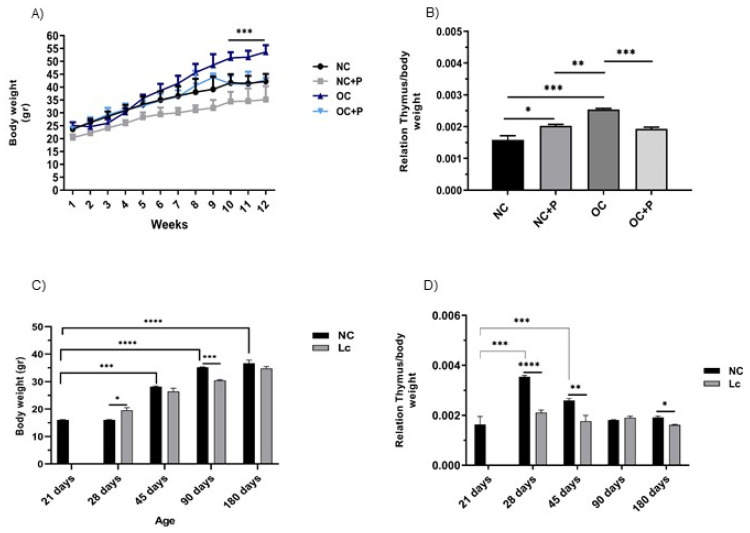
Body and thymus weight changes in senescence and obesity animal models. Mice were weighed every week during the experience. On the day of sacrifice, the thymus weight of each animal was also registered. Each point represented the average ± SD of the weight data pooled from 3 independent experiments with 3 mice per group. (**A**) Body weight differences registered in normal control mice (NC); normal control mice that received a conventional diet and supplementation with the probiotic bacteria (CN+P); obese mice (OC), and obese mice that received the HFD and probiotic supplementation (OC+P). (**C**) Body weight curves of mice at different ages (21, 28, 45, 90 and 180 days of age). The thymus/body weight ratio was calculated by dividing the organ weight by the body weight for (**B**) the group of obese mice and (**D**) mice at different ages. One-way ANOVA with Tukey’s correction for multiple comparisons * *p* < 0.05; ** *p* < 0.001; *** *p* < 0.001; **** *p* < 0.0001.

Normal control mice that received the probiotic suspension showed the lowest weight values during the whole experience, with their weight being significantly lower than the normal control (Figure 1A). Comparatively, the analysis of body weight fluctuations with respect to age showed that, over time, there was a gradual increase in body weight in the animals that received a conventional balanced diet. The animals supplemented with the probiotics showed gradual increases in body weight over time and maintained their weights below those obtained for the normal control (Figure 1C).

At this point, we observed a marked effect on the thymus/body weight ratio in all the mice supplemented with the probiotic strain. OC mice showed the highest values. There was a significant decrease in this ratio in OC+P, reaching similar values to those observed in NC and NC+P (Figure 1B). 

When this relationship was analyzed among the different ages, the highest values were observed at 28 days; then, the values decreased over time. Interestingly, animals that received the probiotics showed a sustained and constant thymus/body weight relationship over time (Figure 1D).

### 3.2. Effect of Probiotic Supplementation on the Intestinal Microbiota

Total populations of enterobacteria, lactobacilli and anaerobes were determined on the large intestine of mice from both experimental models. The enterobacteria population significantly increased in obese mice compared with NC (** *p* < 0.01) and also in old mice compared with younger NC (** *p* < 0.01). Probiotic administration significantly decreased this population in obese mice (* *p* < 0.05) compared with OC. Mice who were 45 and 90 days old consuming the probiotic bacterium showed that their enterobacteria population significantly decreased compared with NC mice at the same age (** *p* < 0.01 for 45 days and * *p* < 0.05 for 90 days). The lactobacilli population significantly decreased in OC with respect to NC (** *p* < 0.01). Interestingly, probiotic supplementation significantly increased this population in obese mice in relation to the obese control (*** *p* < 0.001). Similar results were observed when probiotic feeding was analyzed at different ages. In old mice, probiotic supplementation increased the lactobacillus population at 28, 45 and 180 days in comparison with mice that did not receive probiotic supplementation.

**Figure 2:**
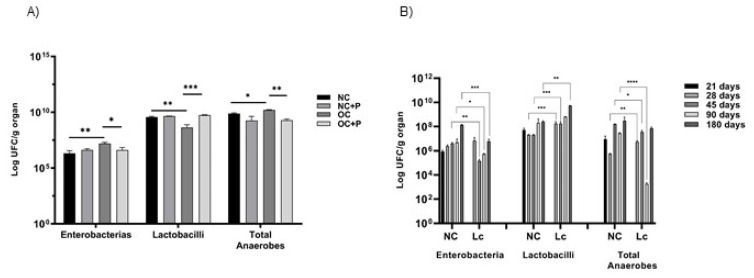
Microbial population in the large intestine. Total population of enterobacteria, lactobacilli and anaerobes was evaluated in the large intestine of: (**A**) obese mice and (**B**) mice of 21, 28, 45, 90 or 180 days for the senescence study. Results were expressed as CFU/mL per gram of large intestine (mean ± SD) of three independent experiments. (NC) received a balanced diet and water; (NC+P) received a balanced diet and probiotic bacteria in the drinking water; (OC) mice fed a high-fat diet and water; (OC+P) mice fed a high-fat diet and a probiotic Lc 431 suspension. One-way ANOVA with Tukey’s correction for multiple comparisons * *p* < 0.05; ** *p* < 0.001; *** *p* < 0.001; **** *p* < 0.0001.

The total anaerobic population increased in both obese and old mice. Probiotic supplementation decreased these values, mainly in 90 day-old mice. These results are shown in Figure 2A,B. 

### 3.3. Histological Studies in Thymus

**Figure 3:**
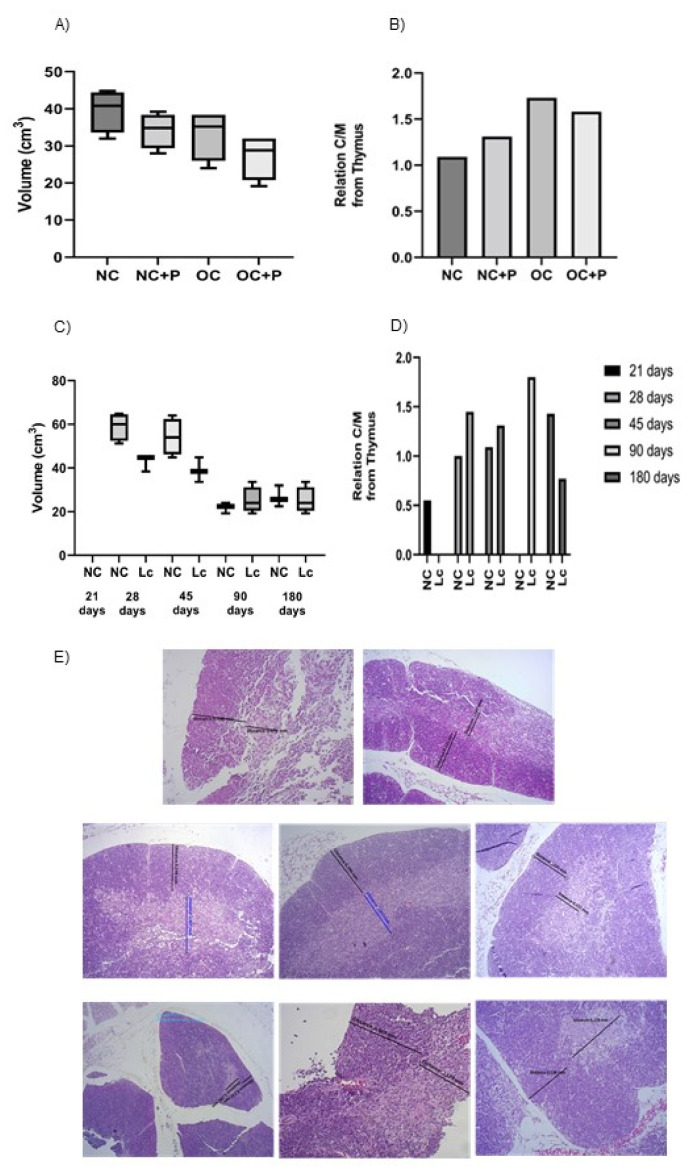
Post-mortem determination of thymus volume. The organ was extracted and weighed after the sacrifice. Each point represented the average ± SD of volume data pooled from 3 independent experiments with 3 mice per group. (**A**,**C**): size of the thymus in the obesity and senescence mouse models, respectively, (**B**,**D**): cortex/medullary /relationship from the thymus in obesity and senescence mouse models, respectively. Results (mean ± SD) were representative of three independent experiments. Micrographs of thymus sections in the different animal models. Tissue sections from obese (**E**) and senescence mouse models stained with hematoxylin and eosin. Magnification 1000×.

At the end of the experiment, the histological slices of the thymus from obese mice showed an important thymus involution compared with the NC group. This effect was evidenced by a reduction in the volume of this organ (Figure 3A). Additionally, a change in the cortex/medullary ratio of the lobules was observed, with a notable decrease in the medullary area at the expense of an increase in the cortex (Figure 3B). Surprisingly, the probiotic administration to obese mice improved the cortex/medullary relationship; however, the volume of the organ remained lower than that observed in the obese control group. 

We observed a clear involution of the thymus with age (Figure 3C,D). Probiotic consumption was not able to reverse the decrease in thymus volume; only a slight increase in thymus size was observed in 90 day-old mice. However, the medullary area was gradually restored with probiotic supplementation. Illustrative photographs of thymus histology are shown in Figure 3E.

### 3.4. Determination of CD4+ and CD8+ T Cell Population in Thymus 

**Figure 4:**
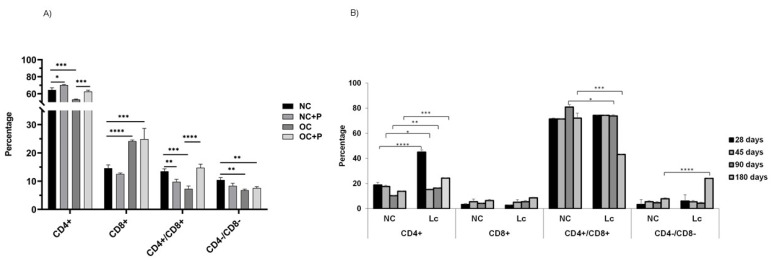
Determination of CD4 and CD8 (single- and double-positive) T lymphocytes in thymus. The number of CD4+ and CD8+ cells was determined by flow cytometry in both models: (**A**) obese mice with or without probiotic supplementation; (**B**) senescence animal model at different mice ages (21, 28, 45, 90 and 180 days) with or without probiotic consumption. Results were expressed as means of the percentages of positive cells obtained from 3 mice per group and repeated in 3 independent experiments. One-way ANOVA with Tukey’s correction for multiple comparisons * *p* < 0.05; ** *p* < 0.001; *** *p* < 0.001; **** *p* < 0.0001.

The analysis of T-cell populations in the thymus of obese mice showed a significant decrease in CD4+ T population with respect to NC (*** *p* < 0.001) (Figure 4A). The same results were obtained in aging mice aged 90 and 180 days old (Figure 4B). The oral supplementation with the probiotic suspension in obese mice increased the percentage of this population compared with OC (*** *p* < 0.001), displaying similar values to those in NC. This increase was also observed in 180-day-old mice receiving the probiotic supplementation. 

In contrast, there was an increase in CD8+ single-positive thymocytes in both obese and older mice with respect to the normal control (*** *p* < 0.001). The administration of probiotics did not modify the levels of this population.

In OC, we observed a significant decrease in CD4+/CD8+ double-positive (DP) thymocytes (*** *p*< 0.001) and CD4−/CD8− double-negative (DN) thymocytes (** *p* < 0.01) with respect to NC. Supplementation with the probiotic suspension in obese mice displayed an increase mainly in DP (**** *p* < 0.0001) and DN thymocytes (Figure 4A). In aging mice, large variations were not observed in these two populations; however, the administration of probiotics induced increases in CD4−/CD8− levels (**** *p* < 0.0001) and a slight decrease in the double-positive population (Figure 4B).

### 3.5. Determination of Cytokine Production by Ex Vivo and In Vitro Studies 

**Figure 5:**
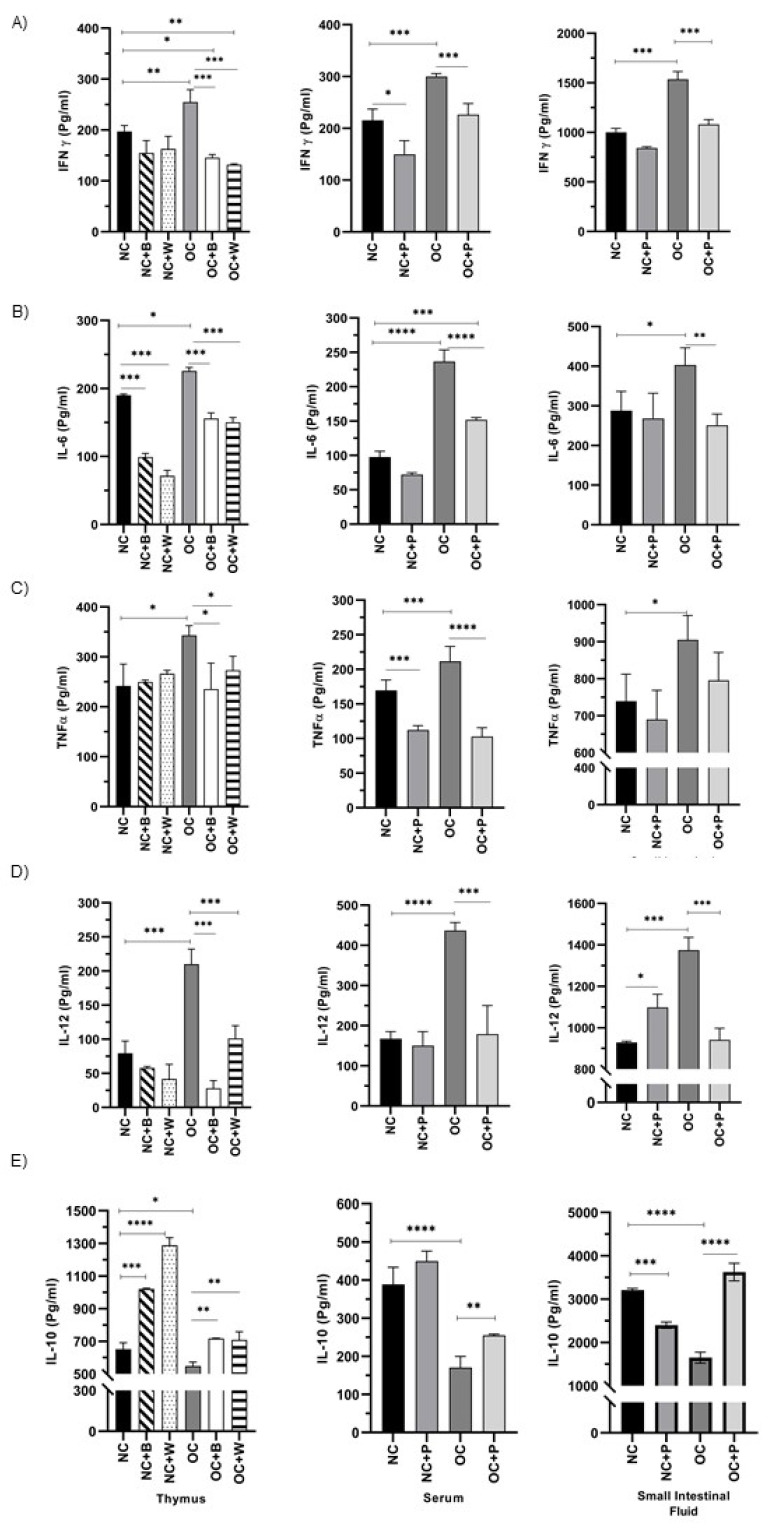
Cytokine production in the obesity mouse model. The levels of different cytokines: (**A**) IFN-γ, (**B**) IL-6, (**C**) TNF-α, (**D**) IL-12 and (**E**) € IL-10 were determined in the thymus, serum, and small intestinal fluid from the animals: NC (normal control); (NC+P) receiving a balanced diet and probiotic bacterium in the drinking water; (OC) fed water and a high-fat diet; (OC+P) fed a high-fat diet and a probiotic suspension with Lc431. In vitro cultures of thymocytes were stimulated with the probiotic bacterium (B) or its probiotic cell wall (W). Data were shown as mean ± SD of 3 independent experiments with 3 mice per group. One-way ANOVA with Tukey’s correction for multiple comparisons. * *p* < 0.05; ** *p* < 0.01; *** *p* < 0.001; **** *p* < 0.0001.

The high-fat diet induced significant increases in the proinflammatory cytokines IL-6, IFN-γ, TNF-α and IL-12 compared with NC mice, not only at the systemic level, but also in the spleen, thymus and the intestinal environment. Oral supplementation with the probiotic suspension decreased the levels of these cytokines in both NC+P and OC+P (Figure 5A–C). When thymus and spleen cells from the OC group of mice were stimulated in vitro with the complete probiotic bacterium or its cell wall, the production of IFN-γ, TNF-α and IL-12 decreased to values close to the NC group. A different behavior was observed in the IL-6 levels produced by spleen cells from OC when they were stimulated with both B and their W, showing an important activation of splenocytes with high IL-6 levels (Figure S2).

The analysis of the regulatory IL-10 cytokine in obese mice showed low levels in serum in relation to NC. Obese mice supplemented with the probiotic suspension tended to increase this cytokine in systemic fluids (serum) (Figure 5A) and the local (small intestinal fluid) environment (Figure 5C). 

In vitro culture of thymus and spleen cells showed a decrease in IL-10 in OC with respect to NC. These cells were stimulated with both the complete bacterium and its cell wall, increasing the levels of this cytokine and normalizing the values (Figure 5B,D).

**Figure 6:**
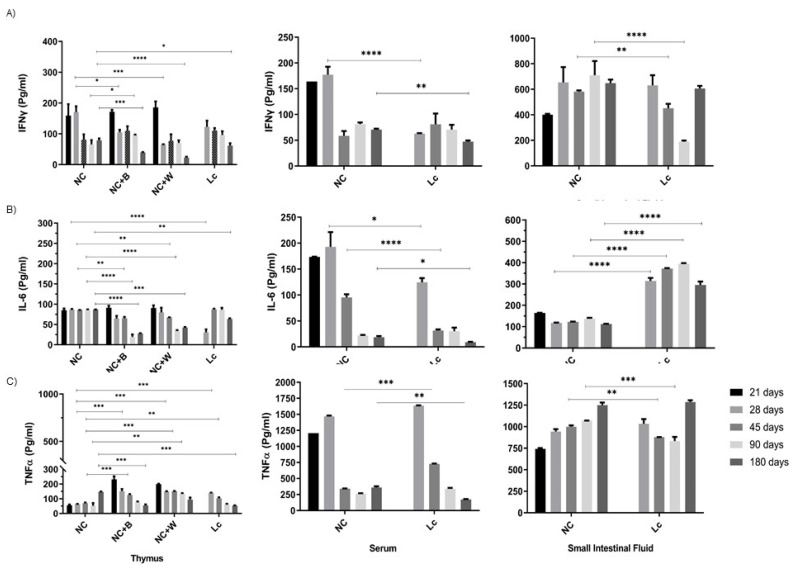
Cytokine production in the senescence mouse model. Levels of (**A**) IFN-γ; (**B**) IL-6; (**C**) TNF-α were determined in the thymus, serum and small intestinal fluid from mice at different ages (21, 28, 45, 90 and 180 days)**.** Thymocytes were in vitro stimulated with the probiotic bacterium (B) or the probiotic cell wall (W), and the cytokines (IFN-γ, IL-6 and TNF-α) were determined in the supernatant collected after 24 h of incubation. Data were shown as mean ± SD of 3 independent experiments with 3 mice per group. One-way ANOVA with Tukey’s correction for multiple comparisons. * *p* < 0.05; ** *p* < 0.01; *** *p* < 0.001; **** *p* < 0.0001.

In the senescence mouse model, we observed that the oldest mice (180 days old) exhibited levels of secreted cytokine (IL-6, IFN-γ, TNF-α and IL-12) below those observed in young animal controls (28 and 45 days old) in splenocyte culture (Figure S3), thymocytes, and serum samples (Figure 6). Administration of the probiotics was not a sufficient stimulus to normalize these values; low levels of cytokines were maintained. 

**Figure 7:**
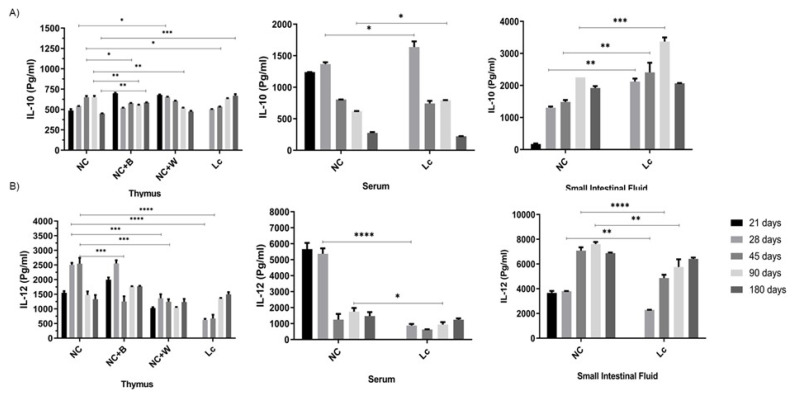
IL-10 and IL-12 production in the senescence animal model. Levels of: (**A**) IL-10 and (**B**) IL-12 were determined in the thymus, serum and small intestinal fluid from mice at different ages (21, 28, 45, 90 and 180 days)**.** Thymocytes were in vitro stimulated with the probiotic bacterium (B) or the probiotic cell wall (W), and the cytokines (IL-10 and IL-12) were determined in the supernatant after 24 h of incubation. Data were shown as mean ± SD of 3 independent experiments with 3 mice per group. One-way ANOVA with Tukey’s correction for multiple comparisons * *p* < 0.05; ** *p* < 0.01; *** *p* < 0.001; **** *p* < 0.0001.

By contrast, intestinal fluids showed high levels of these cytokines in old mice upon probiotic or probiotic cell wall stimulation (Figure 6 and Figure 7B).

In serum, a decrease in IL-10 was also observed in older animals; however, in this case, the probiotic supplementation had no effect (Figure 7A). A positive effect was observed in the thymus and spleen with increases in IL-10 secretion upon the probiotic bacteria or cell wall stimulation (Figure 7A).

**Figure 8:**
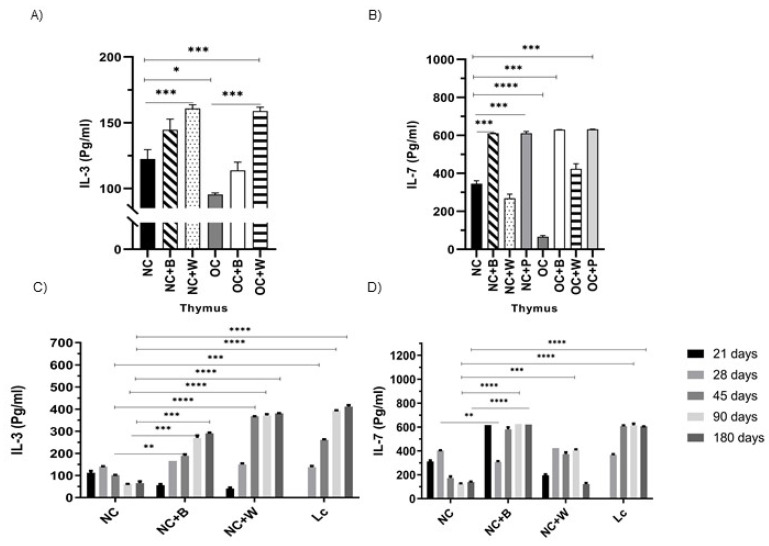
IL-3 and IL-7 levels in thymocytes from obese and old mice. The production of IL-3 (**A**) and IL-7 (**B**) of thymus cells was determined in the thymocyte culture supernatant from normal control (NC) and obese control (OC) mice. In senescent mice, IL-3 (**C**) and IL-7 (**D**) were determined in the thymocyte supernatant from mice at different ages (21, 28, 45, 90 and 180 days). The cell culture was stimulated by the probiotic bacterium (B) or its cell wall (W). Data were shown as mean ± SD of three independent experiments. One-way ANOVA with Tukey’s correction for multiple comparisons * *p* < 0.05; ** *p* < 0.01; *** *p* < 0.001; **** *p* < 0.0001.

We also evaluated the secretion of IL-3 in the thymus cell culture, observing a reduction of this cytokine in OC in relation to NC. Upon stimulation with the complete bacterium or its cell wall, these cells produced an increase in the release of IL-3 in NC and OC (Figure 8A). 

In older mice, a decrease in IL-3 in the thymus was observed, similar to that in obese mice. The probiotic bacterium or cell wall stimulation increased IL-3 above the values obtained for NC (Figure 8C). 

Finally, the levels of IL-7, which play an important role in thymus function, were analyzed. This cytokine showed a significant decrease in the thymus of obese and old mice in relation to NC (Figure 8B,D). Stimulation with the complete bacterium significantly increased the levels of IL-7 with respect to NC and OC. 

## 4. Discussion

Humans and their immune systems undergo a series of age-related changes. One of these age-related changes in the immune system includes thymic involution and Treg accumulation in the aged peripheral secondary lymphoid organs. This condition contributes to immunosenescence and “inflammaging,” defined as a low-grade systemic inflammatory state, which contributes to the dysregulation of various components of the innate and adaptive immune systems [36].

On the other hand, it has been reported that in rodent obesity models, animals display thymus involution and defects in T cell production. This knowledge leads us to think that both conditions, aging and obesity, have many common points that cause immune system deterioration and affect health in this population group.

In the present paper, we analyzed the effect of oral probiotic supplementation on the thymus, considering that this organ is essential for immune maturation. We also evaluated how the probiotic strain could improve immunity in an obese and old host in an experimental animal model.

In obese mice, the HFD induced an increase in body weight by 30% over the normal control, and a reduction in thymus volume was seen with a significant accumulation of peripheral fat. In parallel, in old mice, the thymus showed an important involution, evidenced by a decrease in the thymus/body weight ratio and the volume of this organ. However, histologically, the thymus showed similar characteristics to those observed in the obese population, with increases in the cortical zone with a smaller medullary area. 

The systematic infiltration of fat in the thymus, which occurs over time, replaces hematopoietic tissue, affecting the thymus function and, in consequence, the immune response [37]. The high-fat diet seems to accelerate this process, probably due to the fat accumulation in this organ, which produces disorganization of the tissue with replacement of the functional areas.

The results obtained in this study showed that probiotic supplementation in obese animals recovered histological thymus damage, showing a similar structure observed in the normal control. These results are in concordance with previous ones obtained in obese mice that received a probiotic yogurt [33]. 

We analyzed the thymus T-cell population considering several pieces of evidence that suggest a close correlation between the metabolic and immune systems. In this sense, it is known that in thymus involution, a significant defect in T-cell responsiveness occurs in obese or leptin-deficient mice [38]. A recent study has revealed that there are certain “danger-associated molecular patterns” (DAMPs), such as ceramide and free cholesterol, which start the signaling and induce IL1-β production in the thymic cortex, leading to thymic dysfunction [39]. An HFD induces adipogenesis within the thymus and thymic atrophy, similar to what occurs with aging, characterized by the presence of a large number of adipocytes in the thymus and changes in thymic architecture [40,41]. 

It is thought that interactions of adipocytes with lymphoid progenitors and other stromal cells may have important consequences for thymic biology. Results obtained in this study showed a loss of the normal architecture of the thymus in obese and elderly mice and significant depletion of the lymphocyte population, mainly CD4+ and double-positive T cells. The depletion of the T-cell population, especially the double-positive lymphocytes in the thymus, was reported in Chagas disease, a pathology associated with regression of the thymus and intrathymic and systemic contents of TNF-α during the acute *T. cruzi* infection [42].

The regeneration of the thymus size and function holds great promise for extending the healthspan. Surprisingly, probiotic administration improved thymic cellularity and preserved many immature T-cell precursors in the thymus, even in adult mice and in mice consuming the high-fat diet. These results showed a reactivation of the thymus function after probiotic consumption. Other authors described the same results in old mice that consumed a calorie-restricted diet, suggesting that the quality of the diet is an important factor to maintain immune system surveillance [43]. 

The deficiency of CD4 T cells in the senescence model is accompanied by dysregulated cytokine production [44]. At this point, we highlight the importance of IL-3 production, as it is a cytokine involved in the development and homeostasis of hematopoiesis with the capacity to trigger biologic effects, inducing cell proliferation [45,46,47]. Our results in thymus cultures from obese and old mice showed a positive response in IL-3 production. This allows us to infer that this cytokine is closely involved in the reactivation of the T lymphocytes observed in the thymus. 

Another important cytokine in thymus improvement of animals supplemented with the probiotic bacterium was IL-7, which is produced by stromal and epithelial cells within the thymus. IL-7, a pleiotropic cytokine, plays a key role in lymphopoiesis and lymphoid homeostasis and is essential for early thymocyte development [48,49], lymphocyte survival and expansion [50,51]. 

It is important to highlight that another practical clinical approach for thymic rejuvenation is the use of cytokines, growth factors and hormones. A fusion protein that combines IL-7 and the N-terminal extracellular domain of CCR9 is among the most used. This fusion protein targets the thymus of aging animals, restoring thymus architecture and thymopoiesis [52].

Alterations of the compositional patterns of the gut microbiota have been observed in obesity. There are many investigations that showed an increase in the microorganisms from the phylum Firmicutes and a great decrease in Bifidobacteria [53,54]. A substantial number of reports link the gut microbiota to health in aging. Other studies showed that natural aging decreases the diversity of the microbiota. This is accompanied by a decrease in the bacterial biosynthesis of cobalamin (B12) and biotin (B7), a decrease in the SOS genes that are associated with DNA repair, and a greater degradation of creatine, which is related to muscle atrophy [55].

Elderly people and centenarians also experience a marked decrease in the abundance of the genus Bifidobacterium in the gut, which is related to increased “inflammaging” [56]. Based on these reports, the possibility of improving the intestinal microbiota with dietary supplementation is very attractive. The results obtained with probiotic supplementation improve the intestinal microbiota favoring the Lactobacillus and Bifidobacterial population, which play an important role in normal physiological processes. These microorganisms can synthesize bioactive isomers of conjugated linoleic acid that have antidiabetic, antiatherosclerosis, immunomodulatory, and anti-obesity properties [57]. It has also been shown that lactobacillus fermentation in mouse cecum and human colon leads to the formation of short-chain fatty acids (SCFAs) (lactate, acetate, propionate, and butyrate), with butyrate being one of the major energy sources for enterocytes and colonocytes [58]. Another important effect of butyrate on the gut is its capacity for increasing the expression of tight-junction proteins, reducing the gut hyper-permeability that results in decreased inflammation/endotoxemia associated with the leaky gut [59]. 

It is important to pay attention to the cytokine pattern produced in the thymus microenvironment since their balanced production has a marked influence on thymocyte differentiation, migration and export. In this sense, studies have shown an important correlation between inflammatory cytokine production and severe thymic atrophy with thymocyte migratory disturbances [42]. We analyzed a wide repertoire of cytokines in both old and obese mice, some of which exert pleiotropic effects on different organs. It is important to note that in old mice, cytokine levels generally decrease over time, both in the thymic environment as well as at the systemic and spleen levels, as a consequence of the catabolic process that affects all the cells of the body, including the immune system cells. An important fact was that, at the intestinal level, high levels of inflammatory cytokines were observed. Other authors reported that immunosenescence is associated with “inflammaging”, a chronic low-grade inflammatory status in the intestinal tract, with higher levels of circulating IL-6, IFN-σ, TNF-α, and IL-1 promoting poor fitness and frailty in elderly people [60]. Many of these changes are explained as a consequence of changes in the composition of the human gut microbiota and the slowing of intestinal transit that affects defecation and leads to constipation, inevitably affecting gut homeostasis and the gut-associated immune system [61].

Supplementation with probiotics or in vitro stimulation of culture cells, either with the probiotic bacterium or its cell wall, failed to balance the cytokine levels, especially in older mice, after 90 days. In obese mice, the cytokine study generally yielded opposite results to those obtained in elderly mice. In obese mice, the levels of inflammatory cytokines, TNF-α, IFN-γ, IL-6 and IL-12, were elevated compared to normal mice. Here, probiotics have an effect on the regulation of cytokine profiles.

A relevant aspect derived from our results is that the in vitro stimulation with complete probiotic bacteria or their cell walls, as well as oral supplementation with *L. casei* CRL 431, were able to increase IL-10 levels in the thymus and intestine. This could partly explain the regulation observed in the other cytokines evaluated.

Although it is not established that there is a direct relationship between the intestine and the thymus, scientific evidence allows us to suggest this idea. In previous studies carried out in animal models, we demonstrated that the consumption of probiotics as part of the daily diet had a clear impact on other distant organs from the intestine, such as at the level of the bronchi and mammary glands, with increases in secretory IgA [62].

Other studies conducted in malnourished mice fed with probiotics reported that the histological damage in the thymus induced by the protein-energy imbalance to which the mice were subjected [22] was reversed. 

All these findings allow us to say that intestinal health, in terms of a balanced microbiota and an adequate activation of intestinal immune cells, leads to good immunological surveillance in distant organs from the intestine, such as the thymus. Cytokines play a fundamental role, acting as biological mediators that allow communication between the different organs.

There is not much reported evidence about the use of probiotics regarding thymus recovery. Although many studies are necessary to verify their efficacy, the results obtained here underscore an important thymus-intestine interaction, with many possibilities to continue exploring this area and to scale up to human studies.

## 5. Conclusions

The thymus retains a remarkable capacity to regenerate after the removal of a negative stimulus, which is very promising for healthspan extension. Oral supplementation with probiotic strain Lc 431 has a positive effect on thymus histology that reactivates the function of the thymus with the replacement of the T lymphocyte population. 

Another important aspect is the maintenance of homeostasis in the gut ecosystem, especially during the aging process. The possibility to improve the gut microbiota through dietary manipulation, such as probiotic consumption, results in an interesting perspective for preserving a diverse and healthy gastrointestinal microbial community and improving the regulation of the gut immune system.

## Data Availability

The raw data supporting the conclusions of this article will be made available by the authors without undue reservation.

## Supplementary Materials

The following supporting information can be downloaded at: https://www.mdpi.com/article/10.3390/nu14030616/s1, Figure S1: Study design in senescence and obese; Figure S2: Cytokines production by spleen cells from obese mice. Figure S3. Cytokines production by spleen cells from senescence mice.
