# Supplementary material for: Probiotic Consumption Boosts Thymus in Obesity and Senescence Mouse Models
Source: Nutrients. 2022 Jan 30;14(3):616. doi: 10.3390/nu14030616 (PMC8838891; doi:10.3390/nu14030616)
Supplement: Supplementary file 1 [file nutrients-14-00616-s001.zip › nutrients-1505508-supplementary.pdf]

Supplementary Materials:

A)

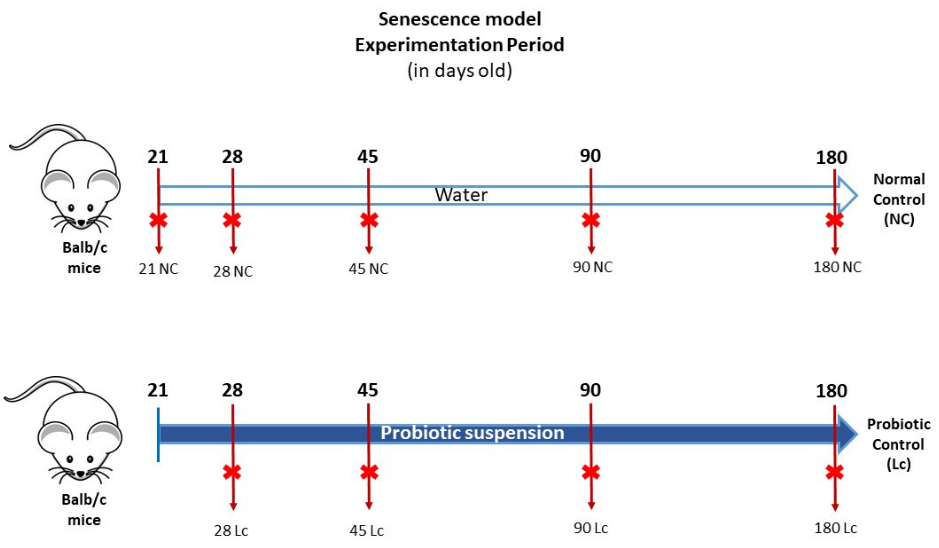

B)

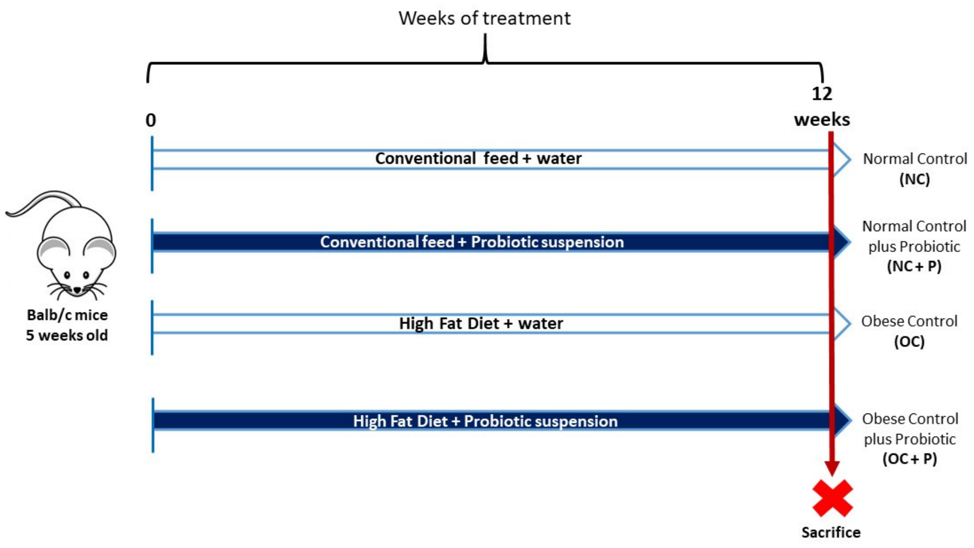

Figure S1. **Study design in senescence and obese mice.** BALB/c mice (21 days-old) were divided in two groups normal control (NC) and probiotic control (Lc) groups. A) Animals of NC groups received a balanced diet and water during all the experience. Mice of PC groups received a balanced diet and the probiotic bacteria Lc431 supplementation in the drinking water. Three mice from each groups were sacrificed at different times 21, 28, 45, 90 and 180 days. B) Mice (6 weeks-old) were divided in four groups: (NC) received a balanced diet and water; (NC+P) received a balanced diet and probiotic bacterium in the drinking water; (OC) fed with water and a high fat diet; (OC+P) fed with

a high fat diet and a probiotic suspension. The animals of each groups were sacrificed after 12 weeks of treatment.

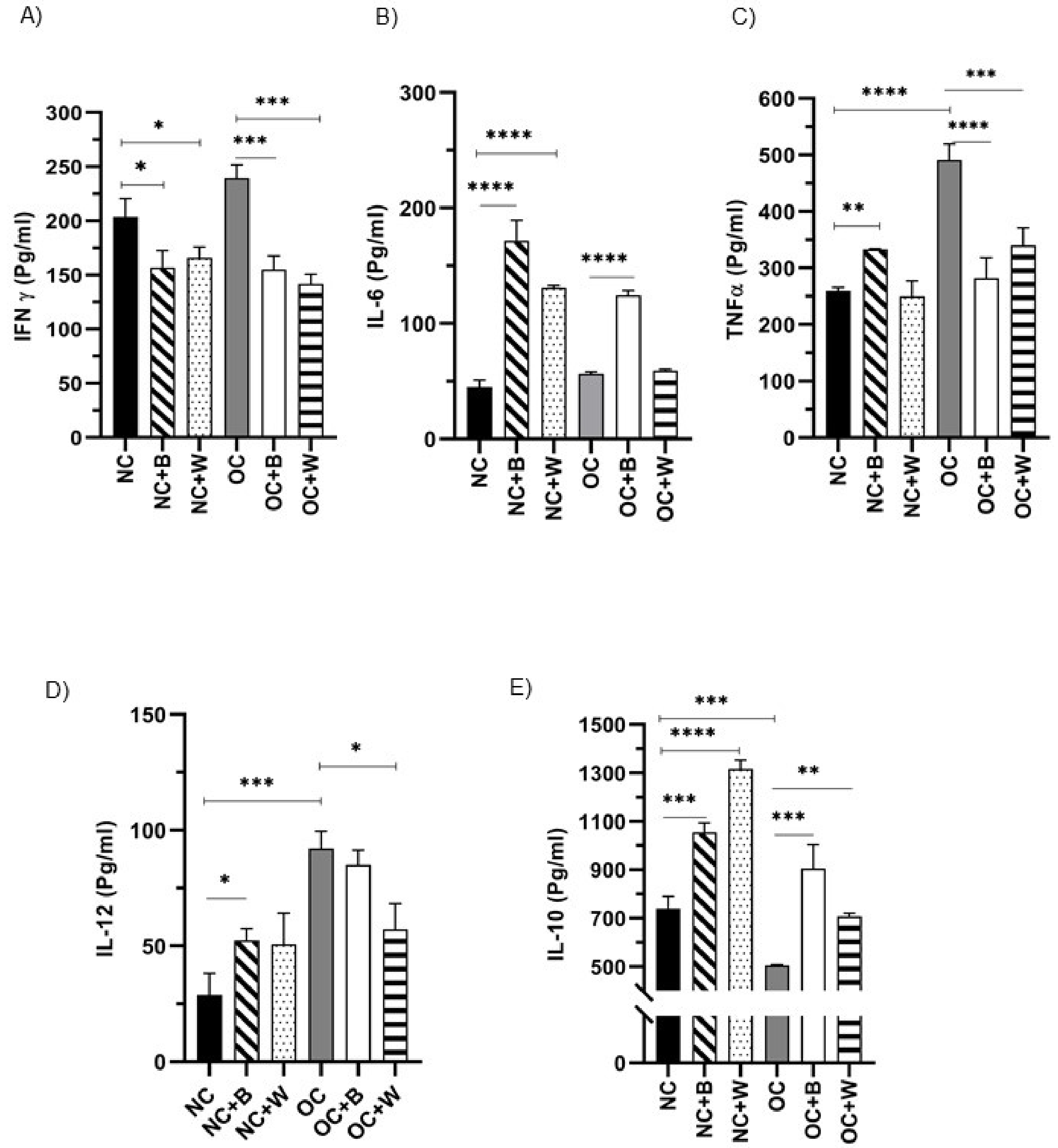

Figure S2. **Cytokines production by spleen cells from obese mice.** The levels of different cytokines: A) IFNγ, B) IL-6, C) TNFα (D IL-12 and E) IL-10 were determined in supernatant of spleen cultures from NC (normal control); (NC+P) received a balanced diet and probiotic bacterium in the drinking water; (OC) fed with water and a high fat diet; (OC+P) fed with a high fat diet and a probiotic suspension with Lc431. Data are shown as mean ± SEM from three independent experiments with 3 mice per group. One-way ANOVA with tukey correction for multiple comparisons. $^*P < 0.05$; $^{**}P < 0.01$; $^{***}P < 0.001$; $^{****}P < 0.0001$. Figure S2.

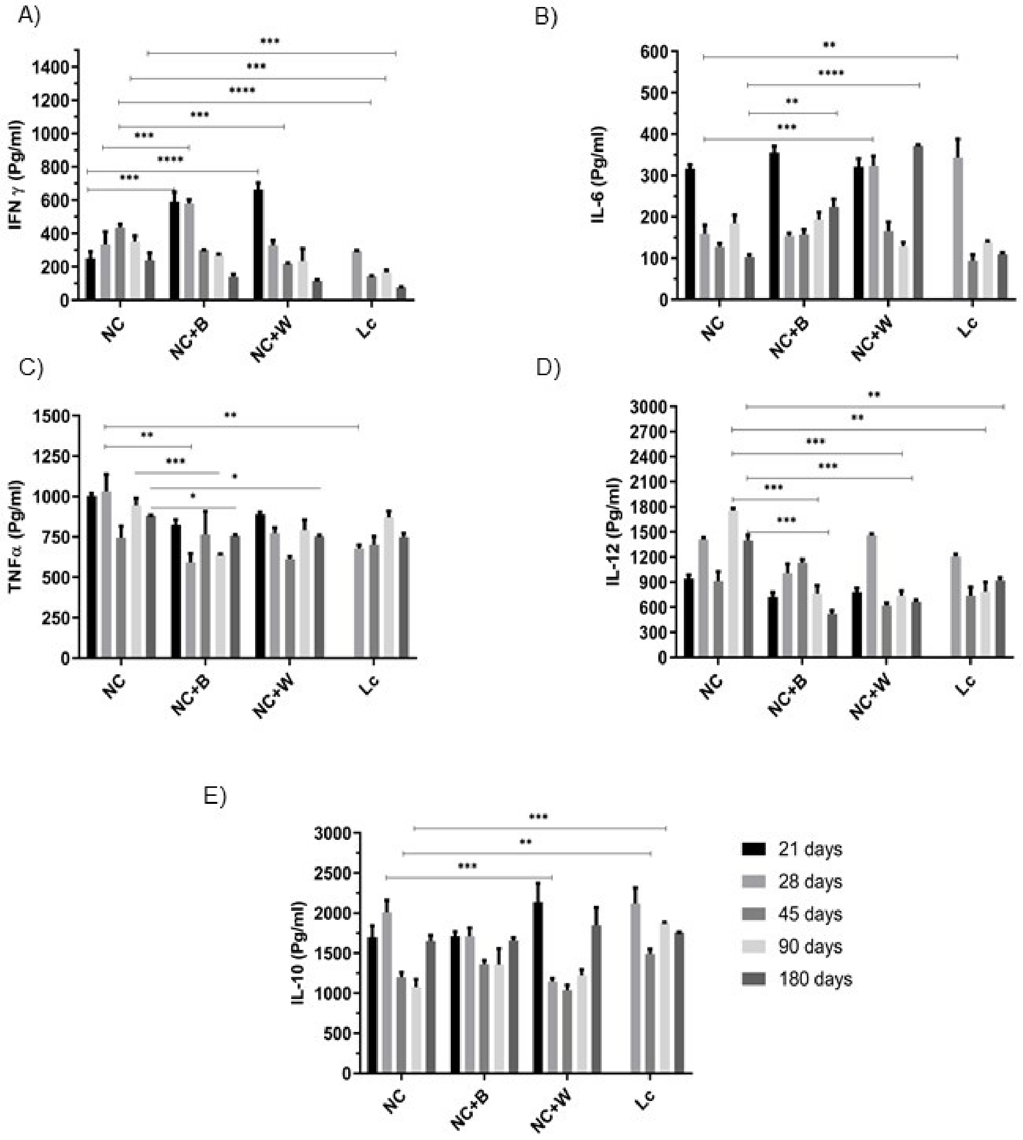

Figure S3. Cytokines production by spleen cells from senescence mice. The levels of different cytokines: A) IFNγ, B) IL-6, C) TNFα (D IL-12 and E) IL-10 were determined in supernatant of spleen cultures from mice at different ages (21, 28, 45, 90 and 180 days). Data are shown as mean ± SEM from three independent experiments with 3 mice per group. One-way ANOVA with tukey correction for multiple comparisons. *$P < 0.05$; **$P < 0.01$; ***$P < 0.001$; ****$P < 0.0001$.
